# Sexual attraction to men as a risk factor for eating disorders: the role of mating expectancies and drive for thinness

**DOI:** 10.1186/s40337-022-00576-z

**Published:** 2022-04-15

**Authors:** Pedro María Ruiz de Assin Varela, Jose Manuel Caperos, Elena Gismero-González

**Affiliations:** UNINPSI Clinical Psychology Center, and Psychology Department, Comillas Pontifical University, Madrid, Spain

**Keywords:** Eating disorders, Body dissatisfaction, Sexual orientation, Epidemiologic studies, Thinness

## Abstract

**Background:**

Men tend to give more importance than women to physical aspects when selecting a partner; thus, the internalization of beauty standards and the ideal of thinness may be greater in populations attracted to men, placing them at a higher risk of eating disorders.

**Methods:**

In a sample (n = 398) of heterosexual and gay men and women, we evaluated the drive for thinness, body dissatisfaction, and bulimic symptomatology. Using ANCOVAs, we analyzed the differences in symptoms score according to sex, sexual orientation and relational status including body mass index (BMI) as covariate; we also evaluated the mediating role of drive for thinness in the relationship between sexual orientation and body dissatisfaction.

**Results:**

We found an increased drive for thinness and body dissatisfaction in men-attracted compared with women-attracted participants; also, body dissatisfaction was greater in women than in men. Heterosexual women presented higher bulimia scores than lesbian women. Gay men open to relationships presented higher drive for thinness and body dissatisfaction scores than those not-open to relationships. Finally, differences in body dissatisfaction between gay and heterosexual men were fully explained by drive for thinness, while, in the case of women, drive for thinness only partially explained these differences.

**Conclusions:**

Attraction to men seems to be a risk factor for EDs in the case of gay men and heterosexual women. In addition, in the case of heterosexual women, other factors independent of the desire to attract men seem to be important.

## Plain English summary

Eating disorders (EDs) are important and common diseases that affect different groups of people differently. Specifically, various studies show a higher prevalence of eating disorders in gay men and heterosexual women. This could be interpreted to be a result of their attraction to men, who tend to place a greater importance on physical attractiveness when looking for a partner. In this study, we compared different ED symptomatology (drive for thinness, body dissatisfaction, and bulimia) in a sample of heterosexual and gay men and women, addressing their relationship with sex, sexual orientation, and relational status. We found an increased drive for thinness and body dissatisfaction in men-attracted (gay men and heterosexual women) compared with women-attracted participants (heterosexual men and lesbian women); also, body dissatisfaction was greater in women than in men regardless of the sexual orientation. Finally, heterosexual women presented higher bulimia scores than lesbian women, and gay men open to relationships presented higher drive for thinness and body dissatisfaction scores than those not-open to relationships (and therefore less inclined to try to attract other people sexually). In summation, our results seem to support the idea that attraction to men seems to be a risk factor for EDs in the case of gay men and heterosexual women, although, in the case of women other factors that are independent of sexual orientation should also be important.

## Background

Eating disorders (EDs) are serious and difficult problems to solve that affect an increasing number of people around the world [1]. Anorexia Nervosa, Bulimia Nervosa, and Binge Eating Disorder are among the fastest growing psychological disorders. The prevalence of these types of disorders in women is two to three times higher than in men [2] but sexual orientation appears to be an important moderator of the relationship between sex and ED prevalence [3]. It has been reported that both clinical and subclinical ED symptoms occur more frequently in sexual and gender minorities compared with their heterosexual and cisgender counterparts [4–6]. On the one hand, bisexual and gay men are considered a risk population for the development of EDs and for presenting isolated or subclinical symptomatology [7, 8]. On the other hand, compared with lesbian women, heterosexual and bisexual women report higher levels of peer appearance pressures [3].

There is an overrepresentation of gay men in clinical samples of EDs [9–11]. The proportion of gay men with ED symptomatology is up to 10 times higher than in heterosexual men [12]. Between 14 and 42% of men seeking treatment for an ED identify themself as bisexual or gay [13]. This overrepresentation does not seem to be due to bias at the time of seeking treatment [14] and is independent of the risk of having other mental disorders [11].

In women, the relationship between sexual orientation and ED risk is less clear [15]. Some studies suggest that lesbian orientation would be a protective factor against these disorders [12], being associated with lower body dissatisfaction [16, 17] and more positive attitudes towards food and weight [18]. Other studies, however, did not find large variations in ED symptoms [13, 19, 20] or body dissatisfaction [21, 22] among women according to sexual orientation. Finally, there are studies that even consider that minority sexual orientation, far from being a factor of protection, can come to represent an added risk in women. Some studies have found that lesbians have a higher frequency of purging behaviors [23], bulimic symptoms [24], and higher levels of obesity and overweight [25].

Regardless of their sexual orientation, men place more importance on physical appearance when selecting a partner, while women place greater emphasis on other factors, such as personality, status, power, or income [26]. Siever [27] hypothesized that gay men and heterosexual women are dissatisfied with their bodies and vulnerable to EDs because of a shared emphasis on physical attractiveness and thinness, based on a desire to attract and please men. In turn, lesbians and heterosexual men should be less concerned with their own physical attractiveness and, consequently, less dissatisfied with their bodies and less vulnerable to EDs. If attraction to men is a key factor in the understanding of ED vulnerability [14, 28–30], two implications may be expected. First, the relationship status should moderate the relation between sexual orientation and ED symptomatology: those who are open to or looking for new relationships will present greater symptomatology [14, 29]. For example, in heterosexual women, marriage is associated with lower ED symptoms [31, 32] or, gay single men score higher in food symptomatology than those who maintain stable relationships [33]. Second, if gay men and heterosexual women vulnerability to EDs roots on the shared emphasis on physical attractiveness and thinness [27], the degree of internalization of the thinness ideal should mediate the relationship between sexual orientation and ED symptomatology. In this sense, although the drive for muscularity has been found to be a beauty ideal in the case of men, it is usually accompanied by worry about body weight or leanness [34–36], and the internalization of the thin-ideal was associated with body dissatisfaction in both men and women [37].

The first objective of the study was to evaluate the relationship of ED symptomatology with sex and sexual orientation. Specifically, we assessed this relationship for each ED component: drive for thinness, body dissatisfaction, and bulimia. We would expect a larger proportion of symptoms in men-attracted participants (heterosexual women and gay men). Second, if the interest in attracting men is associated with EDs, we would expect the relationship status to moderate ED symptoms, especially in high-risk populations (heterosexual women and gay men). Lastly, we aimed to explore the role of drive for thinness in the mediation of the relationship between sexual orientation with body dissatisfaction and bulimic symptomatology.

## Method

### Sample

The study sample comprised 398 participants (*n* = 170, 42% men) with a mean age of 29.48 (SD = 4.12; Min = 20; Max = 35). Forty-five percent of the men (n = 77) and 68% of the women (n = 159) reported to be sexually attracted to men. Men’s mean BMI was 24.0 (SD = 2.8; Min = 17.3; Max = 36.1), and women’s mean BMI was 22.2 (SD = 3.4; Min = 16.8; Max = 42.2) (Table 1). Of the sample, 46.5% (*n* = 195 participants) declared themselves to be open to relationships either because they were single (*n* = 159) or because they were involved in a non-exclusive relationship (*n* = 36). Data was collected by an online questionnaire disseminated via social network. In this process, to increase the number of participants of less frequent sexual orientations, the questionnaire was sent via sexual and gender minorities associations and collectives. The instructions to participants avoided the use of terms such as EDs, disorder, or symptom, asking them to participate in an investigation on the relationship of some sociodemographic and psychological variables with eating habits. They were also informed that the collected data was completely anonymous, and that the questions did not include personal information that could allow respondents to be identified. The questionnaire was answered by 419 participants, from this first sample, we excluded 15 participants (three men and twelve women) attracted to both sexes, and six (five men and one woman) who declared no sexual attraction.

**Table 1:** Descriptive data of sample in function of sex and sexual orientation

Sex	N	Age (SD)	BMI (SD)	Singles^†^	Mated^†^
*Attracted to*					
Men					
Men	77	29.7 (3.8)	23.1 (2.4)	33/0	15/29
Women	93	30.4 (4.2)	24.7 (2.8)	42/5	5/41
Women					
Men	159	28.6 (4.2)	22.0 (3.4)	45/7	6/101
Women	69	30.0 (3.6)	22.7 (3.6)	34/1	7/27

^†^Open/ not open to a new relationship

### Variables and measures

Each participant was asked about their sex, age, weight and height, to calculate the BMI. Sexual orientation was measured by the statement: “I am usually sexually attracted to…” which could be completed by answering *Men*, *Women*, *Both* or *Neither*. In a pretest study, this formulation was found to be better received than the 'Straight'/'Homo'/'Bi'/'Asexual' options because it did not involve identifying with a label that many volunteers experienced as restrictive. This is consistent with findings from the preceding literature [15]. Four alternatives were included instead of two ('Heterosexual'/'Gay' or 'Heterosexual'/'Other') similar to other studies [14, 29], in order not to assimilate participants with very different identities into the same group.

We measured ED symptomatology via the Spanish version of the Eating Disorders Inventory-2 [38, 39]. The EDI questionnaire measures 11 dimensions of ED symptomatology using a 6-point Likert scale (0 = Never—5 = Always). Item responses were coded following test instructions, the most extreme response in the anorexia direction (always or never depending on the keyed direction) earning a score of 3; the immediately adjacent response 2, the next response 1 and the three choices opposite to the anorexia direction receiving no score. Scale scores were calculated as the sum of all item scores for that scale. In this study we considered only three of the subscales: drive for thinness, body dissatisfaction, and bulimia, those reported to be more directly related to ED symptomatology [14]. The three scales presented good internal consistency in the study sample: drive for thinness (α = 0.98), body dissatisfaction (α = 0.98), and bulimia (α = 0.98).

Relationship status was assessed by two questions: First, participants were asked if they were in a stable relationship, and then if they were open to establishing a new relationship (sexual or emotional). This was carried out to collect the variability corresponding to people who, being in a relationship, were willing to have relationships with others, and to register those who, being single, showed no interest in establishing sexual relationships. For the aim of the study, the important point was not whether the person was involved in a relationship, but whether they were interested in attracting new partners, given the higher rate of open relationships or nonconsensual nonmonogamy reported in several study populations of heterosexual men, gay or lesbian [40]. Thus, the open or not open to a new relationship dichotomous variable was the variable considered in the analysis.

### Data analysis

To assess differences in each EDI-2 scale, we ran an ANCOVA including drive for thinness, body dissatisfaction or bulimia scores as the dependent variable, sex and attraction as fixed factors, and BMI as covariate. Then, to explore the moderation effect of the relationship status, we explored differences in symptomatology according to relationship status for each sex by attraction orientation group separately via ANCOVA, including BMI as covariate. Finally, to evaluate the mediation role of drive for thinness in the relationship of attraction with body dissatisfaction, we ran a mediation model including attraction as factor, drive for thinness as mediator, and body dissatisfaction and bulimia as response variables. Eta-squared (*η*^2^) was reported as effect size in the case of ANCOVA although, when we specifically aimed to describe the differences between groups, we reported standardized mean difference as Cohen's *d*. Analyses were performed using Jamovi [41] and SPSS [42], with a confidence level of 95%.

## Results

**Fig. 1:**
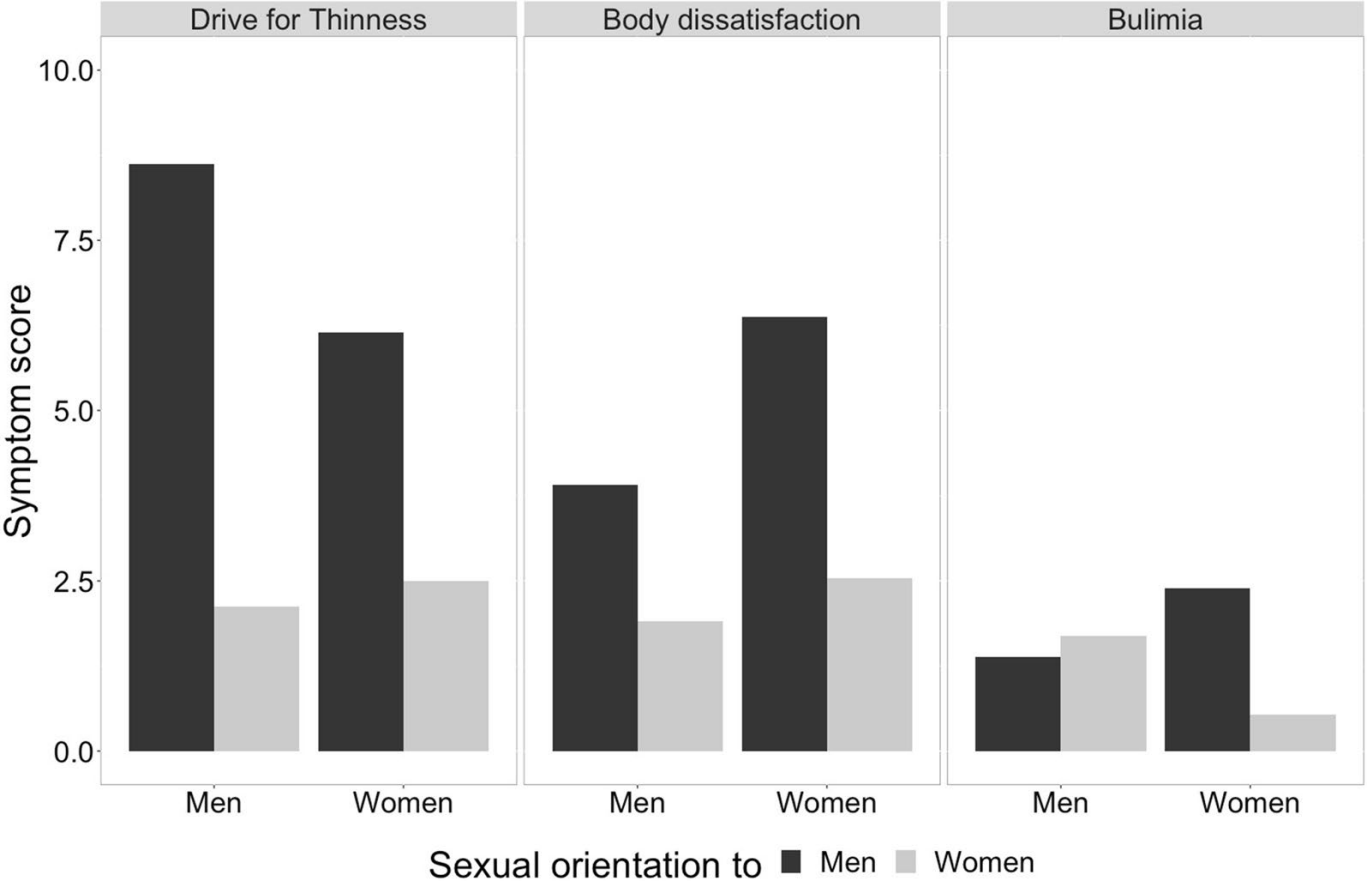
Drive for thinness, body dissatisfaction, and bulimia scores in function of sex and sexual orientation

As expected, we found that men-attracted participants presented higher drive for thinness than participants attracted to women, *F*(1, 393) = 76.50; *p* < 0.001; *η*^2^ = 0.163 (Fig. 1). We also found a significant interaction between sex and sexual orientation, *F*(1, 393) = 6.28; *p* = 0.013; *η*^2^ = 0.016, the difference in drive for thinness was significatively larger between gay and heterosexual men (*d* = 1.13; *p* < 0.001) than between lesbian and heterosexual women (*d* = 0.67; *p* < 0.001). We did not find a main effect of sex *F*(1, 393) = 1.90; *p* = 0.168; *η*^2^ = 0.005.

In the case of body dissatisfaction, men attracted participants scored more than women attracted ones, *F*(1, 393) = 40.60; *p* < 0.001; *η*^2^ = 0.094, and, also, that women presented greater body dissatisfaction than men, *F*(1, 393) = 16.57; *p* < 0.001; *η*^2^ = 0.040. We did not find any interaction between both variables, *F*(1, 393) = 1.97; *p* = 0.161; *η*^2^ = 0.005.

Men-attracted participants presented higher bulimia scores than women-attracted ones, *F*(1, 393) = 8.61; *p* = 0.004; *η*^2^ = 0.021. Although, an interaction occurred between sex and sexual orientation, *F*(1, 393) = 12.47; *p* < 0.001; *η*^2^ = 0.031, this difference was only significant in the case of women (*d* = 0.63; *p* < 0.001), not for men (*d* = 0.12; *p* = 0.705). We did not find a main effect of sex *F*(1, 393) = 0.04; *p* = 0.848; *η*^2^ = 0.000 (Table 2).

**Table 2:** Mean and standard deviation for men and women in function of sexual orientation and symptom

	Sexual orientation					
	To men		To women		Total	
	Mean	DT	Mean	DT	Mean	DT
Drive for thinness						
Men	8.6	7.6	2.1	3.5	5.1	6.6
Women	6.2	5.9	2.5	4.3	5.0	5.7
Total	7.0	6.6	2.3	3.8	5.1	6.1
Body dissatisfaction						
Men	3.9	5.2	1.9	3.3	2.8	4.4
Women	6.4	5.9	2.5	4.9	5.2	5.9
Total	5.6	5.8	2.2	4.1	4.2	5.4
Bulimia						
Men	1.4	2.3	1.7	2.8	1.6	2.6
Women	2.4	3.4	0.5	1.5	1.8	3.1
Total	2.1	3.1	1.2	2.4	1.7	2.9

Separate analysis per sex-attraction orientation group showed that gay men scored higher for body dissatisfaction and drive for thinness when they were open to a new relationship than when not, and that lesbian women scored lower for body dissatisfaction when open to a new relationship than when not. We did not find differences in the case of heterosexual men nor women (Table 3).

**Table 3:** Differences in symptomatology in function of the relationship status, sex and sexual orientation

Symptomatology	Open to a relationship			Not open to a relationship			Differences between relationship status groups			
	*N*	Mean	SD	*N*	Mean	*SD*	*F*	*dF*	*p* value^†^	*d*
Gay men										
Drive for thinness		12.67	6.61		1.93	3.22	65.66	(1, 74)	< 0.001	1.92
Body dissatisfaction	48	5.15	5.89	29	1.86	3.09	7.78	(1, 74)	0.021	0.65
Bulimia		1.29	1.74		1.55	3.13	0.23	(1, 74)	1.00	− 0.11
Heterosexual men										
Drive for thinness		1.34	2.94		2.93	3.91	3.26	(1, 90)	0.222	− 0.46
Body dissatisfaction	47	1.19	1.91	46	2.63	4.16	2.58	(1, 90)	0.336	− 0.44
Bulimia		1.26	2.50		2.13	2.96	1.25	(1, 90)	0.801	− 0.32
Heterosexual women										
Drive for thinness		6.69	5.89		5.90	5.93	0.45	(1, 156)	1.00	0.13
Body dissatisfaction	51	6.90	6.00	108	6.13	5.92	0.27	(1, 156)	1.00	0.13
Bulimia		2.82	3.84		2.19	3.13	1.18	(1, 156)	0.837	0.19
Lesbian women										
Drive for thinness		1.95	4.18		3.29	4.34	1.79	(1, 66)	0.558	− 0.31
Body dissatisfaction	41	1.10	2.44	28	4.64	6.67	9.44	(1, 66)	0.009	− 0.76
Bulimia		0.34	0.94		0.82	2.02	1.61	(1, 66)	0.624	− 0.32

^†^*p* values are adjusted for three comparisons by Bonferroni correction

Finally, we ran a mediation analysis to evaluate whether drive for thinness is a mediator in the relationship between sexual orientation and body dissatisfaction. We could not run this model in the case of bulimia because differences in bulimia and drive for thinness did not occur in the same direction. As the relationship between sexual attraction and drive for thinness is different between men and women, we included sex as a moderator in the mediation analysis.

Results of the mediation analysis showed that, while in the case of men, the drive for thinness fully mediated the relationship between sexual attraction and body dissatisfaction, in the case of women, this mediation was only partial and sexual orientation had a direct effect on body dissatisfaction, not explained by the drive for thinness component (Table 4).

**Table 4:** Mediation effects of drive for thinness in the relationship between sexual orientation and body dissatisfaction

Sex	Effect^†^	Estimate	SE	*Β*	*z*	*p*
Average						
	SO ⇒ DT	− 0.725	0.084	− 0.410	− 8.64	< 0.001
	DT ⇒ BD	0.414	0.028	0.601	14.67	< 0.001
	SO ⇒ BD	− 0.044	0.052	− 0.036	− 0.85	0.396
Men						
	SO ⇒ DT	− 0.928	0.122	− 0.524	− 7.57	< 0.001
	DT ⇒ BD	0.318	0.028	0.488	11.28	< 0.001
	SO ⇒ BD	0.072	0.074	0.063	0.98	0.326
Women						
	SO ⇒ DT	− 0.523	0.115	− 0.295	− 4.56	< 0.001
	DT ⇒ BD	0.510	0.028	0.684	18.06	< 0.001
	SO ⇒ BD	− 0.160	0.066	− 0.122	− 2.42	0.015

^†^*SO* sexual orientation, *DT* drive for thinness, *BD* body dissatisfaction

## Discussion

Regarding the first aim of the study, and as previously described in the literature [7, 8, 12, 16], heterosexual women and gay men presented greater ED symptomatology than lesbian women and heterosexual men, but these differences depended on the specific symptomatology evaluated. In the case of drive for thinness, differences appeared in the men-attracted group, and, in this group, symptomatology was greater in men than women; Body dissatisfaction was greater in men-attracted participants regardless of their sex but was also higher in women regardless of their sexual orientation; Bulimia symptomatology was much greater in heterosexual than in lesbian women and was similar to men, regardless of their sexual orientation.

As proposed by Siever [27], ED risk may be partially driven by mating motivations. Given that men place more importance on body shape and physical features than women when choosing potential partners [26], people attracted to men may be under greater pressure to have an attractive physique, focusing on those aspects that are mainly considered to be attractive. The thin-ideal is typically the most widespread Western ideal of beauty [43]; and the internalization of thin-ideal places men-attracted people at risk of body dissatisfaction symptomatology. In recent years, the use of social media has been widely related to the exposure and internalization of such ideals [44–48]. Social networks affect the self-image of both men and women and, therefore, their idealized content serves as a comparison criterion for both sexes [49].

Gay men presented the highest drive for thinness scores of the four groups, higher than heterosexual women, suggesting a particular vulnerability to thin-ideal internalization. Some studies reported that bisexual and gay men are more susceptible than heterosexual men to social messages focusing on physical appearance [50] and place more importance on physical appearance and attractiveness [51]. Notably, Gigi et al. [50] found that they showed increased attention to social comparison information, increased internalization of cultural ideals presented in the media about appearance, and susceptibility to the influence of advertisements that emphasize appearance, which was interpreted as a consequence of interest to please other men. In this regard, Li et al. [30] reported that, when faced with a context of intrasexual competition, gay men reported more restrictive eating attitudes and more body image concerns than in non-competitive scenarios or than heterosexual men. According to the authors, contexts of intrasexual competition should elicit desires to be especially thin for many individuals given that intrasexual competition is established in relation to those characteristics that are desirable.

In the case of body dissatisfaction, on the one hand, we found a main effect of sexual orientation. Body dissatisfaction was higher in gay men than in heterosexual men, and also in heterosexual woman than in lesbian women. He et al. [52] analyzed 75 primary studies published between 1986 and 2019, finding that sexual minority men had a higher level of body dissatisfaction than heterosexual men (57 studies, 128 effect sizes), with small to medium effect sizes. This result partially support Siever’s view, as well as suggesting that the internalization of thin-ideal by women and gay men places men-attracted people at risk of body dissatisfaction symptomatology. Meanwhile, on the other hand, we also found a main effect of sex, with women presenting higher scores than men, regardless of their sexual orientation. In general, women are under greater sociocultural pressure of an aesthetic ideal and most studies reported a higher prevalence of body dissatisfaction in women [53]. Women, for example, have lower body satisfaction than men regardless of their BMI [54]. Up to 80% of women respondents expressed current dissatisfaction with their bodies [55], and body dissatisfaction was found to be the most potent predictor of EDs [56]. Social pressures mean that women see their appearance as a fundamental factor in their value as individuals and expect others to routinely examine them [57]. This may lead them to scrutinize their body image, increasing the risk of being dissatisfied with it [58, 59].

In the case of bulimia, lesbian women reported the lowest bulimia scores, being much lower than heterosexual women, while for men there were no differences based on sexual orientation. Higher body dissatisfaction was found to be a predictor of bulimia [53]. Gay men also presented high levels of body dissatisfaction; however, this did not translate into a high rate of reported bulimia symptomatology. Given that gay men still emphasize the importance of a lean but also athletic physique, with a low percentage of body fat [51, 60, 61], it seems logical that they would seek to minimize behaviors contrary to that ideal. In this sense, some authors already propose that for men, sexual attraction to men would only be a risk factor for restrictive eating symptomatology [14, 29]. Although there are others who continue to find significant differences in measures of bulimia or impulse regulation [33].

Lesbian women presented the lowest bulimia symptomatology. This result is in line with other studies suggesting that, in women, a lesbian sexual orientation would be a protective factor against EDs [12], being associated with lower body dissatisfaction [16, 17]. However, similar research has yielded contradictory results: Dotan et al. [62], in a recent meta-analysis examining the association between sexual orientation and disordered eating in women, reported that there was no significant difference in overall disordered eating between lesbians and heterosexual women, however, lesbians reported restricting less and bingeing more than heterosexual women.

The second aim of our study was to evaluate the effect of relationship status on ED symptomatology. According to Siever’s hypothesis, a greater symptomatology would be expected when looking for/or open to a new relationship than when monogamously mated. As expected, we found that gay men presented higher scores for body dissatisfaction and drive for thinness if they were looking for/or open to new romantic or sexual partners. This result would indicate a greater attention/concern to the evaluation of their own physical appearance when looking for partner. As Siever [27] suggested, gay men pursuing a partner suffer increased pressure to be physically attractive, and those who feel they do not meet the high attractiveness ideals of the gay community may experience heightened body image concerns, as they feel their bodies may not be appealing enough to attract a partner. Men in relationships may be less exposed to objectifying experiences within the gay culture compared with single men actively pursuing partners and putting themselves in environments where such pursuits may occur, such as in gay clubs or on dating websites and apps [63]. As Parker and Harriger [64] pointed out, among sexual minority men, the use of dating apps was found to be an ED risk factor, which is likely due to the added pressure to adhere to a certain aesthetic to attract more potential sexual partners. Brown and Keel [65] showed that, although it made no difference to heterosexual men, bisexual and gay men who were single had an increased drive for thinness, and that being in a relationship may be a protective factor for body image concerns and disordered eating among gay men. Cella et al. [33] also found gay sexual orientation associated with greater body dissatisfaction and abnormal eating behaviors in men, especially among those who were not in a sentimental relationship. All these findings support the notion that single men may be more concerned with their appearance than those in relationships; our results also support that gay men are at higher risk when they are open to finding a new partner.

In contrast, we did not find an effect of relationship status on the symptomatology of heterosexual women. As stated previously, Siever’s [27] hypothesis does not seem to apply fully in the case of women. This result is consistent with other studies that found that the symptomatology of lesbian women was not always less than that of heterosexual women [62], and that explanatory models of ED etiology followed similar patterns in lesbian and heterosexual women, but not in heterosexual and gay men [66]. Our mediation analysis pointed suggestively in the same direction. While in men the differences in body dissatisfaction derived from sexual orientation were fully explained by their drive for thinness, in the case of women, thinness only partially explained body dissatisfaction. Thus, the thin-ideal seem to fully explain differences in body dissatisfaction between heterosexual and gay men, but not completely between heterosexual and lesbian women.

The cognitive component of body dissatisfaction has been considered to contain two categories, preoccupation with the body and self-objectification on the one hand, and internalization of the thin-ideal, on the other [43]. Objectification theory [57] posits that girls and women are acculturated to internalize an observer’s perspective as a primary view of their physical selves, and that women could respond to sexual objectification in function of sexuality, age, ethnicity, and other physical and personal attributes. Cultural objectification can be internalized as self-objectification leading to increased body monitoring and body dissatisfaction [67–69]. Therefore, differences between lesbian and heterosexual women may lie not only in the direction of the attraction, but in a different internalization of gender roles [70, 71]. Gender role adoption is related to body dissatisfaction and EDs [72] and femininity has been considered a more critical factor than sexual preference on ED psychopathology [73]. The comparison of women with different sexual orientations could be involving two different issues, differences in the orientation of their attraction (mediated by the interiorization of beauty ideals), and differences in the process of socialization. This would explain both the inconsistency of results found in the literature on the relationship between sexual orientation and EDs in the case of women, as well as the results of the present study.

This study has several limitations. First, given the split of the sample into groups of sex, sexual orientation, and relationship status, some of these groups presented small sample sizes and the associated tests might have low statistical power. Likewise, the absence of significant results in some groups should be taken with caution. Despite this, descriptive measures of effect size seem to point in the direction of our conclusions. Second, we use the drive for thinness measure as a measure of the degree of internalization of thin-ideal, which does not fully correspond. However, different studies show a positive relationship between the two measures [74–76], therefore, we consider that the conclusions derived from the results are appropriate. Finally, while aiming to evaluate risk factors, the cross-sectional nature of data collection does not allow us to follow the evolution of the symptoms or to distinguish in the mediation models the antecedent variables from their outcomes.

## Conclusions

In sum, sexual orientation seems to be a risk factor for EDs in gay men. Also, in gay men, body dissatisfaction seems to be mediated by the internalization of the thin-ideal and related to the search for a partner. The puzzle seems more complex in the case of women. While some of our results suggest that lesbian sexual orientation may be a protective factor for EDs in women (bulimia), others indicate a specific risk due to the fact of being a woman (body dissatisfaction). Body dissatisfaction in the case of heterosexual women does not seem to be completely mediated by the internalization of the thin-ideal or related to the search for new relationships. Our results seem to indicate that while attraction to men is a main risk factor for EDs in the case of gay men, in the case of heterosexual women, in addition to attraction to men, other cultural factors must be considered.

## Data Availability

The datasets used and/or analyzed during the current study are available from the corresponding author on reasonable request.
